# Kounis Syndrome as First Manifestation of Allergic Sensitization

**DOI:** 10.1155/2019/6317956

**Published:** 2019-06-25

**Authors:** D. Forlani, G. Scarano, A. D'Alleva, M. Di Marco, L. Paloscia, A. Gatta, L. Della Valle, A. Farinelli, A. Lumaca, C. Petrarca, R. Paganelli, L. Di Giampaolo, M. Di Gioacchino

**Affiliations:** ^1^Intensive Coronary Unit and Interventional Cardiology, “Spirito Santo” Hospital, Pescara, Italy; ^2^Immunotoxicology and Allergy Unit, CeSI-Met, G. d'Annunzio University, Chieti, Italy

## Abstract

Mast cells are abundant in the heart, among myocardial fibers, around coronary arteries, within arterial intima and intramural vessels, and in atherosclerotic plaques. Their mediators can be released during anaphylaxis and be responsible for acute coronary syndrome. This condition has been described as Kounis syndrome (KS). We report three cases of acute myocardial ischemia, which fulfill the definition for KS. In Cases 1 and 2, the association of intense chest pain with acute urticaria after an allergenic contact (wasp sting and betalactam antibiotic administration, respectively) was suspected to be an attack of angina related to an allergic reaction. No signs of an allergic reaction were observed in Case 3, but only the history of a wasp sting suggested its relationship to loss of consciousness and heart ischemia when hypersensitivity to venom was ascertained. These cases strongly recommend measurement of anaphylactic biomarkers, such as tryptase, during acute coronary syndromes to detect the possible involvement of an allergic reaction. Conversely, measurement of cardiac biomarkers during anaphylaxis, even without obvious signs of myocardial ischemia, might identify patients at risk of myocardial injury.

## 1. Background

Systemic anaphylaxis, the most dramatic clinical presentation of allergy, represents a medical emergency in which cardiac and peripheral vascular symptoms dominate the clinical picture and are often the leading cause of death. Cardiovascular manifestations include hypotension and shock, arrhythmias, ventricular dysfunction, cardiac arrest, and cardiac ischemia. The latter is essentially due to the coronary blood flow impairment during anaphylaxis and may contribute significantly to an unfavorable outcome. Mast cells, whose degranulation is the first event of an allergic reaction, are present in the human heart mainly between myocardial fibers, around blood vessels, and in the arterial intima. They are particularly abundant in atherosclerotic plaques [1, 2]. If anaphylaxis occurs, pre-existing coronary artery disease is considered a negative prognostic factor. Activated cardiac mast cells have the capacity to degranulate and release large quantities of mediators, both preformed (histamine, tryptase, chymase, and carboxypeptidase A) and *de novo* synthesized (cysteinyl leukotriene C4, prostaglandin D2, and platelet-activating factor) [1, 3]. Mediators released locally by heart-resident mast cells and eosinophils recruited to the site and activated during anaphylaxis can directly damage the myocardium leading to ischemia that has been described as Kounis syndrome (KS). KS is classified into three subtypes according to the condition of coronary arteries: the first with normal vessels, the second with coronary atheromasia, and the third with coronary thrombosis already treated with stent for previous thrombosis [4, 5].

In the present study, we report 3 cases of Kounis syndrome and discuss its pathophysiology.

## 2. Case Reports

### 2.1. Case 1

**Figure 1:**
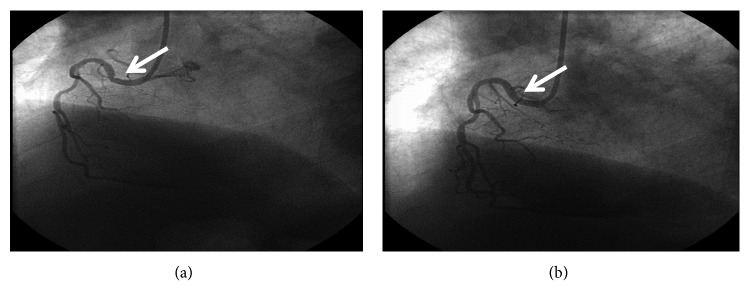
(a) Right coronary angiography documenting coronary spasms at the proximal tract. (b) Angiography performed after intracoronary administration of isosorbide dinitrate documenting coronary spasm resolution.

A 62-year-old man was admitted to the emergency department with intense chest pain and acute urticaria occurring a few minutes after a wasp sting on the right shoulder. No cardiovascular risk factors or history of allergy were recorded. Blood pressure was 90/60, heart rate was 100 b/m, and no signs of hemodynamic instability were found at presentation. The ECG showed ST-T elevation in D2-D3-aVF. The echocardiogram on admission showed normal cavity geometries with mild depression of the left ventricular function at 50% ejection fraction (EF) and akinesia of the lower wall. Myoglobin was 362 ng/mL (normal range < 110), Ck-MB 5.13 ng/mL (normal range < 5.0), and troponin 0.32 ng/mL (normal range < 0.04). Eosinophilia (765 *μ*l) and basophilia (288 *μ*l) were present. ASA 250 mg and sodium heparin 5000 IU/e.v., ticagrelor 180 mg/orally, and methylprednisolone 40 mg/e.v. were given for the emergency treatment of coronary syndrome and urticaria. Coronary angiography showed a significant stenosis of the right proximal coronary artery (Figure 1(a)), due to coronary spasm, as the administration of isosorbide dinitrate 2 mg induced rapid resolution of the stenosis (Figure 1(b)), remission of angina, and normalization of the altered ECG. The patient was discharged on the third day and referred to the allergy unit. Skin tests and specific IgE showed sensitization to wasp venom. Tryptase was 15 mcg/l. Specific immunotherapy for wasp venom was prescribed, and it is currently administered.

### 2.2. Case 2

**Figure 2:**
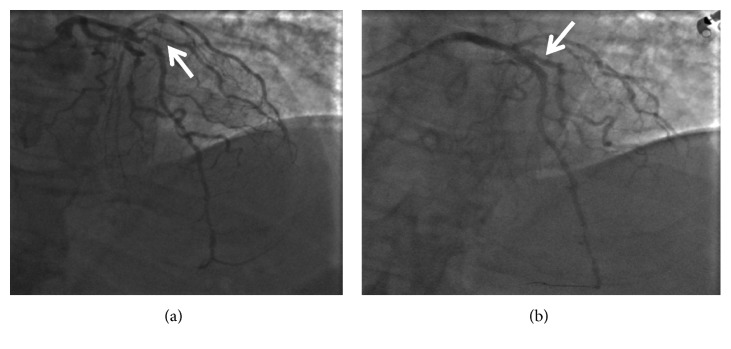
(a) Coronary angiography showing the presence of thrombosis of the middle segment of the AIV coronary artery. (b) Resolution of critical stenosis.

A 61-year-old man developed urticaria shortly followed by loss of consciousness a few minutes after intravenous infusion of ceftriaxone while undergoing surgery (long saphenous vein stripping) under local anesthesia. He was transferred to the emergency department with cardiogenic shock and ECG signs of myocardial anterior ischemia (ST-T elevation in the anterior leads). Blood cardiac biomarkers were normal, but they suddenly increase in laboratory tests 4 hours later (troponin = 16.2 ng/mL, myoglobin = 1103 ng/mL, and Ck-MB = 97 ng/mL). Coronary angiography showed the presence of thrombosis of the middle segment of the anterior interventricular coronary artery causing subocclusion and ischemia (Figure 2(a)). The patient presented cardiovascular risk factors as obesity and hypertension, but no history of cardiovascular disease.

Treatment consisted in emergency primary PTCA and drug-eluting stents of the anterior interventricular artery followed by PTCA and stenting in election on the coronary arteries affected by critical stenosis (Cx and Cdx), with resolution of the subocclusion (Figure 2(b)). ECG signs of ischemia normalized on the third day, with left anterior hemiblock and no Q wave. An echocardiogram showed mild hypertrophy of the left ventricle and hypokinesia of the interventricular septum and the apex. Ejection fraction was 50%. The patient was discharged (with prescription of ASA 100 mg/day, clopidogrel 75 mg/die, and atorvastatin 80 mg/die) and referred to the Allergy Unit, where a diagnosis of ceftriaxone allergy was made (intradermal test positive at 1/100 dilution in physiological saline, specific IgE to cefaclor (Thermo Fisher antigen c7) = 2.1 kUA/l).

### 2.3. Case 3

A 60-year-old man was admitted to the Emergency Room in unconscious state, with signs of hemodynamic impairment. An ECG showed ST-T depression in the inferior and anterolateral leads. Blood levels of cardiac biomarkers were elevated. The patient presented cardiovascular risk factors (dyslipidemia and smoking) and suffered a stroke in 2017. He was immediately treated with steroids and epinephrine. Since his blood pressure fell to a low level of 65/45 mmHg, he was put on norepinephrine until a systolic blood pressure of 95 mmHg was reached. An ECG showed ST-T elevation in the anterior leads. Troponin was 17.6 ng/mL, myoglobin 1341 ng/mL, Ck-MB 92 ng/mL, and WBC count 22,650 cells/uL; eosinophils and basophils were normal. Coronary arteriography showed a subocclusion of the anterior descending branch of the left coronary artery (Figure 2(a)).

**Figure 3:**
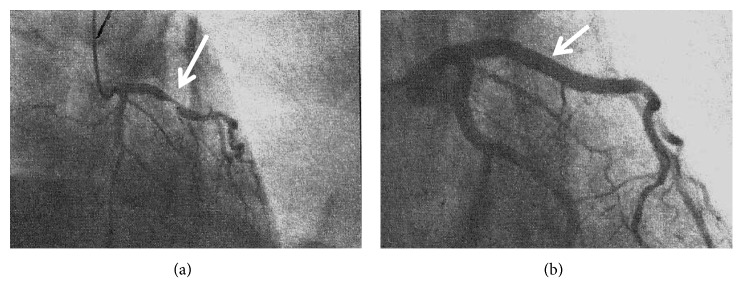
(a) Coronary arteriography showing subocclusion of the anterior descending branch of the left coronary artery. (b) Resolution of the subocclusion.

Treatment consisted of emergency primary PTCA and drug-eluting stents of the proximal and middle segment of the anterior interventricular artery, with resolution of the subocclusion (Figure 3(b)). On the third day, an echocardiogram showed normal cavity geometries and wall thickness of the left ventricle, good global kinetic, and EF 55%. Blood levels of troponin and Ck-MB were reduced. The patient was discharged from the hospital with ASA 100 mg/day, ticagrelor 90 mg 2 bid, bisoprolol 1.25 mg/day, atorvastatin 80 mg/die, and ramipril 2.5 mg/day and referred to the general practitioner. A careful medical history revealed that immediately before losing consciousness the patient was stung by a wasp; following the diagnosis of hymenoptera allergy (positive skin prick test and specific IgE to wasp), the patient started a desensitizing therapy.

## 3. Discussion

Case 1 represents KS type I, characterized by a coronary spasm in normal coronary arteries, in the absence of predisposing factors. The first descriptions of Kounis syndrome focused on patients with “angiographically” normal coronary arteries, in which acute release of allergic mediators could induce vasospastic angina. Cases 2 and 3 represent Kounis syndrome type II, characterized by acute allergic reactions followed by acute myocardial infarction with angiographic evidence of coronary artery disease. In this variant, quiescent atherosclerotic plaques may rupture, and coronary thrombosis may develop as a result of the acute release of inflammatory mediators by resident mast cells after an allergic stimulus. KS type III includes patients already treated with stent for coronary thrombosis in which the activation of mast cells, abundant in atherosclerotic lesions, induces release of mediators, in particular, platelet-activating factors [6], causing the stent thrombosis [7].

Moreover, also the presence of a localized hypersensitivity reaction was a risk factor for the occurrence of late stent thrombosis, as confirmed by the RADAR (Research on Adverse Drug Events and Reports) project that showed the association among local hypersensitivity reaction, thrombosis, and lack of intimal healing after drug-eluting stent (DES) deployment [3]. For all cases described in the RADAR project, DES-induced hypersensitivity reactions were associated with nonurticarial rash, hives, dyspnea, myalgia/arthralgia, itching, and blisters.

The clinical presentation of Kounis syndrome varies widely from an arterial spasm without necrotic myocardial enzyme release up to acute infarction.

In all our cases, the patients were not aware of their sensitization, and the cardiac manifestation was the first anaphylactic event. In Case 1, chest pain occurred shortly after the wasp sting and in Case 2 immediately after ceftriaxone infusion (most cardiovascular events due to anaphylactic shock occur within 60 minutes, with the most frequent involvement of the left ventricular wall).

In Case 1, eosinophilia and basophilia were observed on admission, and a slight increase in serum tryptase persisted up to 1 month after discharge. Elevated baseline concentrations of mast-cell tryptase and mastocytosis are potential risk factors for severe allergic reactions to hymenoptera venom [8], and a threshold level of mast-cell derived tryptase and chymase has been reported, above which coronary artery spasm and/or plaque erosion or rupture is induced [4, 5]. At present, the pathogenesis of KS can be explained by the effects of mast-cell mediators on the heart. Histamine, usually responsible for vasodilation and permeabilization, may induce a decrease in coronary blood flow and severe spasm of large coronary arteries, acting on H1 receptors; moreover, it induces tissue factor expression in human endothelial and vascular smooth muscle cells [9, 10]. Of the other mediators, leukotrienes cause a rapid and sustained increase in coronary vascular resistance and vasoconstriction, thus reducing myocardial perfusion [11]; thromboxane promotes platelet aggregation and vasoconstriction [12]; and the platelet-activating factor delays atrioventricular conduction, decreases coronary blood flow, and induces platelet activation and aggregation, leading to thrombosis [13, 14].

Tryptase is known for activating metalloproteinases in the blood vessel walls, degrading collagen caps, and inducing plaque erosion and rupture, which also leads to the exposure of endothelial prothrombotic molecules [15]. Heart-resident mast cells release an unusually large quantity of chymase, compared to lung or skin mast cells [16]; chymase converts angiotensin I to angiotensin II with vasoconstricting effects [17].

The event should be simultaneously treated for acute coronary syndrome and allergic reaction, but careful attention should be paid to the use of adrenaline, as this may lead to a worsening of coronary vasospasm, described as Tako-tsubo cardiomyopathy [4, 18].

Beyond the acute episode, prognosis of KS is good in the majority of cases, with left ventricular function normalizing in a matter of weeks. Mortality rate is unknown, essentially due to shock, pulmonary edema, and arrhythmias [4].

KS may have several causes, including allergy (to drugs, food, latex, hymenoptera, and anisakiasis) (Table1) [1] and conditions such as scombroid syndrome, Churg–Strauss eosinophilic polyangiitis, and systemic mastocytosis [4, 5].

However, because of the evidence of high levels of tryptase and/or histamine in patients suffering from acute coronary events unrelated to an allergic reaction [2, 5], it is not universally accepted that KS is always due to anaphylaxis. Also, raised tryptase levels are observed in coronary artery disease as a result of chronic low-grade inflammatory activity present in the atherosclerotic plaques [19, 20], suggesting a possible role of tryptase in plaque stability. However, there is evidence that mast-cell activation does not occur in coronary spasm since tryptase increases in patients undergoing a spontaneous episode of myocardial ischemia, but not during an ergonovine-induced coronary spasm [18, 21].

The occurrence and severity of anaphylactic episodes with heart and coronary involvement suggest the usefulness of measuring cardiac biomarkers in subjects with anaphylaxis, also in the absence of symptoms related to heart ischemia, in order to promptly identify and manage potential myocardial injury. In fact, “silent” (asymptomatic) myocardial ischemia is a common manifestation of coronary heart disease [22]. Conversely, serum levels of tryptase should be measured in all patients with acute coronary syndrome in order to reveal a possible allergic etiology. This is particularly important in the absence of coronary obstruction on angiography. Unfortunately, tryptase has a short half-life (3 h), which may prevent detection in clinical practice. In patients with associated signs and symptoms of urticaria, angioedema, or dyspnea, a link to an allergic trigger may be suspected. Protection of the cardiac tissue and the emergency treatment of the acute ischemic episode should be combined with the identification and treatment of the allergic sensitization, the cause of anaphylaxis, to prevent further episodes.

## Figures and Tables

**Table 1:** 

Allergens reported as responsible for Kounis syndrome	
Drugs	
Antibiotics	Amoxicillin, cephalosporins, ciprofloxacin, clarithromycin, clindamycin, metronidazole, penicillins, telithromycin, vancomycin
Anticancer drugs	Cisplatin, oxaliplatin, cyclophosphamide, 5-fluorouracil, rituximab
Antiviral	Brivudine, oseltamavir
NSAID	Aspirin, diclofenac, ibuprofen, methimazole, propyphenazone
Steroids	Hydrocortisone, methylprednisolone, triamcinolone
Used in anesthesia	Propofol, etomidate, midazolam, rocuronium, atracurium
Others	5-Fluororacil, atropine, clopidogrel, enalapril, astemizole, fluconazole, gelofusin, hyoscine butylbromide, insulin, lanzoprazole, oral contraceptive pills, nafamostat, pseudophed, ranitidine, sodium bicarbonate, tramadol, ziprasidone
Insects	Bee, black widow spider, scorpion, snake, wasp
Foods	Blue crab, crucian, kiwi, mushroom, rice, shell fish, tuna fish, salt fish, uncooked anchovies
Others	*Anisakis*, dialysate, latex, intravenous contrast media
